# Endovascular Downstaging: A New Method for Managing Renal Cell Carcinoma Tumor Thrombus Invading the Inferior Vena Cava Above the Hepatic Veins (Level III) or into the Heart (Level IV)

**DOI:** 10.3390/cancers17020264

**Published:** 2025-01-15

**Authors:** John A. Libertino, Malik Ahmed, Thomas Piemonte, Jason Gee

**Affiliations:** 1 Department of Urology, Tufts University Medical Center, 800 Washington St., Boston, MA 02111, USA; jgeemd@gmail.com; 2 Department of Cardiology, Lahey Medical Center, Burlington, MA 01805, USA; malik.ahmed@lahey.org (M.A.); thomas.c.piemonte@lahey.org (T.P.)

**Keywords:** endovascular downstaging, renal cell cancer, IVC tumor thrombus, cardiopulmonary bypass, circulatory arrest, renal cancer level III and level IV

## Abstract

Background: Renal cell carcinoma tends to invade venous structures, frequently extending beyond the inferior vena cava and into the heart itself, such as into the right atrium or right ventricle. Resection of tumor burden, particularly tumor thrombus, often requires cardiopulmonary bypass (CPB) and deep hypothermic circulatory arrest (DHCA), which is not feasible for all patients. Methods: Described in this study is a novel, minimally invasive endovascular approach involving endovascular thrombectomy as a viable approach in these select patients. Results: There were no surgical complications, shorter operating times, less blood loss and an average length of stay of 5.5 days in the four patients undergoing this procedure. Conclusions: We demonstrate that this technique can eliminate the need for cardiac bypass and deep hypothermic cardiac arrest and its associated risks, thereby making surgery safer and more accessible for patients with advanced kidney cancers with an inferior vena cava tumor thrombus. Furthermore, it allows for this life-saving surgery to be carried out in medical centers or hospitals where cardiac surgery is unavailable, or when cardiopulmonary bypass is medically contraindicated.

## Simple Summary

Kidney cancers can sometimes form a tumor thrombus capable of growing into the renal vein and inferior vena cava, and these tumor thrombi can even extend above the diaphragm and into the right atrium and right ventricle. Surgical removal of these tumor thrombi, along with the kidney tumor, is necessary to cure patients, but can require cardiac bypass and circulatory arrest, which adds significant risk for patients undergoing surgery. Here, we describe a novel technique of endovascular tumor thrombus removal prior to surgery. We demonstrate that this technique can eliminate the need for cardiac bypass and its associated risks, thereby making surgery safer and more accessible for patients with advanced kidney cancers with a venous tumor thrombus extending into the inferior vena cava and above the diaphragm, as these patients would otherwise need to undergo cardiac bypass with circulatory arrest for curative surgery.

## 1. Introduction

**Figure 1:**
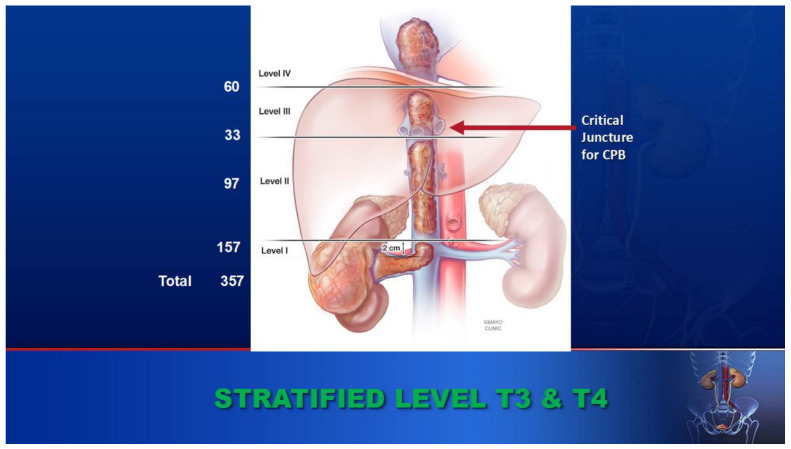
Our experiences of tumor thrombus occurrence at level I–IV (Neves classification).

A unique feature of renal cell carcinoma (RCC) is its predilection to involve venous structures. A tumor can grow within the lumen of the renal vein and propagate into the inferior vena cava up to and beyond the right atrium. This occurs in 4–10% cases of RCC [1]. We have used the Neves classification [2] to stratify the level of the tumor thrombus. Figure 1 outlines the anatomic distribution and the total number of patients we cared for at each level I–IV, including 93 patients with level III and level IV tumor thrombus (Figure 1).

The objective of surgical management of renal cell carcinoma with tumor thrombus extending into the inferior vena cava (IVC) is the complete resection of all tumor burdens. In addition to radical nephrectomy and tumor thrombectomy [3,4,5,6,7,8,9,10], this surgery may also require the resection of the IVC, with or without reconstruction, and a retroperitoneal lymph node dissection.

**Figure 2:**
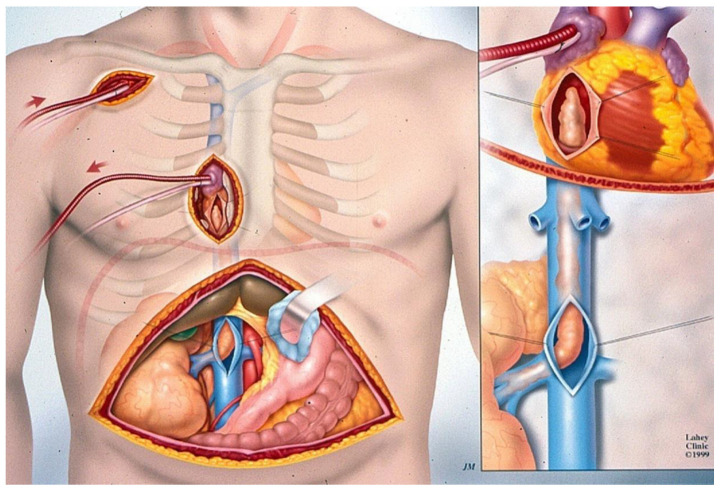
DHCA and minimal access CPB approach.

Management of tumor thrombi requires careful preoperative planning, and execution remains a technical challenge, usually requiring cardiopulmonary bypass (CPB) and deep hypothermic circulatory arrest (DHCA). Initially, in our institution, we had approached these level III and IV tumors by utilizing the median sternotomy approach [11,12,13,14,15], but currently, we favor the minimal access DHCA and CPB approach (Figure 2) [16,17]. A total of 70 of the 93 patients with level III and level IV thrombi have been treated with deep hypothermic cardiac arrest (DHCA) and cardiopulmonary bypass (CPB) [18].

We present a new concept of endovascular downstaging as an alternative to cardiopulmonary bypass and circulatory arrest for the management of level III and level IV tumor thrombus.

## 2. Materials and Methods: Endovascular Downstaging Technique

Patients initially selected to undergo endovascular downstaging had a renal tumor with either a level III or IV tumor thrombus and would otherwise be candidates for radical nephrectomy with IVC tumor thrombectomy with CPB and DHCA. Preoperative staging by MRI and CT scans were obtained for all patients. Patients with metastatic disease were eligible for postoperative treatment, whether by surgical resection of oligometastatic disease or systemic treatment.

Endovascular downstaging was conceived out of necessity for the first patient we treated who had no other safe option for the removal of a level IV tumor thrombus. He was a 54-year-old male with no significant medical history, who presented with night sweats, dizziness, and 50-lb unintentional weight loss over a 1-year period. On evaluation, he was found to have a right renal mass with an inferior vena cava (IVC) tumor thrombus. Prior to surgery, the patient sustained a massive pulmonary embolism requiring catheter-directed thrombolysis.

He was then referred for surgery and a preoperative transesophageal echo cardiogram (TEE) was obtained to reassess the level of his tumor thrombus. This study demonstrated the thrombus extended from the IVC, through the right atrium and right ventricle to the level of the pulmonary valve.

In this setting of a recent massive pulmonary embolism, the patient was not a candidate for cardiopulmonary bypass (CPB) for fear of a very substantial cytokine release following DHCA and CPB. The interventional cardiologist was then consulted for an attempted endovascular thrombectomy, as no other practical alternative existed.

**Figure 3:**
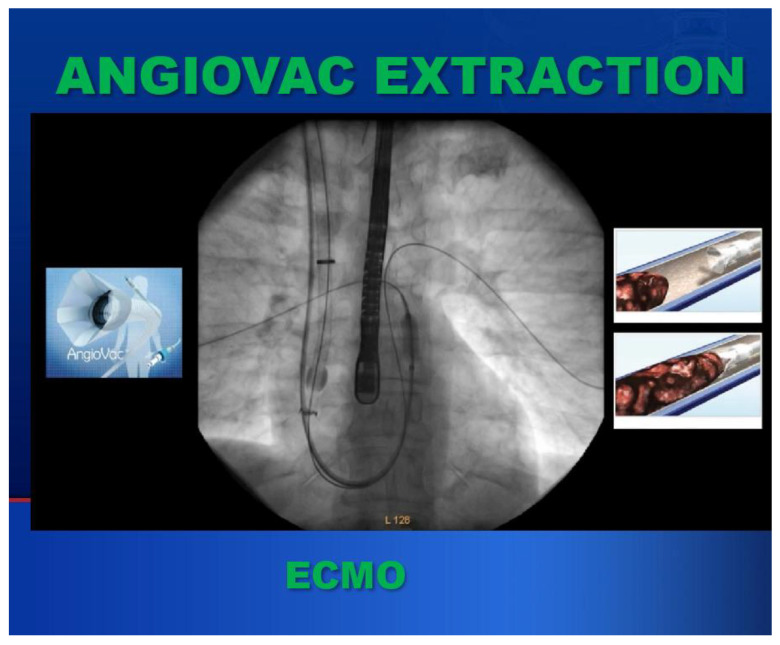
Images of the downstaging technique.

**Figure 4:**
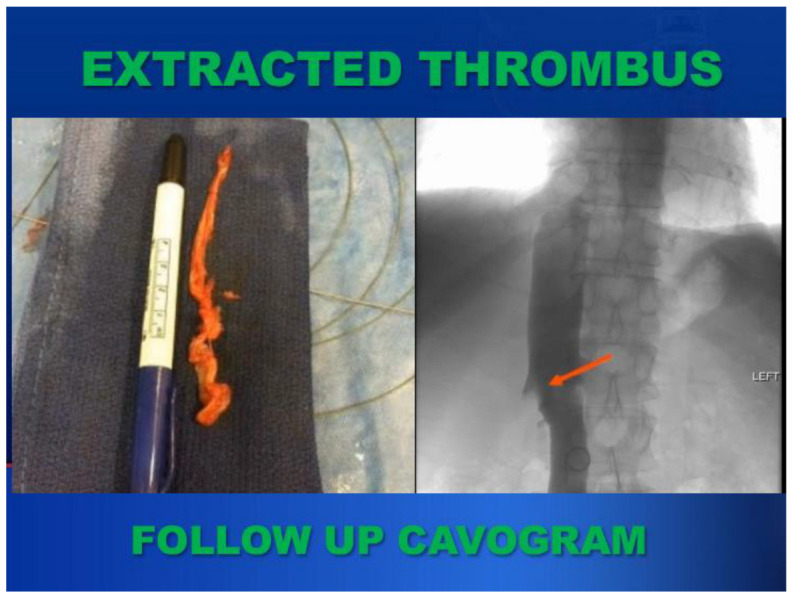
Extracted thrombus and postoperative cavogram. Arrow: remaining residual thrombus after endovascular downstaging.

Using the AngioVac System, the interventional cardiologist successfully extracted the ventricular and IVC thrombus under TEE guidance (Figure 3). The technique is described in detail below. The pathology of the specimen demonstrated clear cell carcinoma. A post-extraction cavogram demonstrated a small residual tumor thrombus at the renal vein ostium (Figure 4). This procedure effectively down staged the tumor from a level IV to a level I thrombus.

To our knowledge, when we originally reported this technique, this was the first description of an endovascular extraction of an intracardiac tumor thrombus [19].

At the time of this report, three additional patients with level III or IV tumor thrombi were also treated by endovascular downstaging prior to surgery in a similar manner. The sample size in this series precludes statistical analysis. The relevant preoperative clinical data for all four patients are included in Table 1.

## 3. Sequence of the Downstaging Procedure

The downstaging procedure is carried out under general anesthesia by the interventional cardiologist, and consists of the following steps, from imaging to percutaneous transvenous mechanical thrombectomy:

1. Placement of a transesophageal echocardiogram (TEE) probe prior to the procedure is essential for image guidance and continuous monitoring.
2. The right internal jugular or common femoral vein are accessed percutaneously, using a modified Seldinger technique.
3. An occlusive balloon catheter is inserted into the main pulmonary artery to act as a retroactive vascular control for the prevention of pulmonary embolism.
4. An AngioVac System (Angio Dynamics; Latham, NY, USA), which is a mechanical aspiration system with a 22-Fr coil-reinforced venous drainage cannula with a balloon-actuated, proprietary expandable funnel-shaped distal tip intended for the removal of thrombi or emboli once the balloon is activated, is utilized to assist with keeping the cannula free of unwanted intravascular material while allowing for the collective removal of thrombi or emboli. The AngioVac system is inserted into the inferior vena cava or into a cardiac chamber after therapeutic anticoagulation, with the insertion point determined by the level of the thrombus and performed under transesophageal echocardiographic guidance.

The tumor thrombus is subsequently evacuated to a level below the hepatic veins. In a subsequent surgical procedure, the resection of the remaining tumor thrombus and radical nephrectomy is accomplished.

In the one patient with the right ventricular thrombus, extra corporeal membrane oxygenation (ECMO) was added to the procedure [19].

## 4. Results

We retrospectively reviewed the results of four patients, three males and one female, with level Ill and level IV tumor thrombi who had endovascular downstaging as an alternative to cardiopulmonary bypass and circulatory arrest. Two patients had level III, and two patients had level IV thrombus prior to endovascular downstaging. Furthermore, two patients had synchronous metastatic disease, whereas the other two developed metachronous metastases.

After endovascular downstaging, all patients subsequently underwent a radical nephrectomy, lymph node dissection, and IVC thrombectomy, which included cavotomy with reconstruction. Reconstruction consisted of the primary closure of the cavotomy with a running 3-0 prolene suture.

At the time of surgery, patients had an average age of 68 years (with a range from 54 to 84 years). Preoperative conditions included: massive pulmonary embolism (PEx2 pts), pulmonary metastases, myocardial infarction (MIx2 pts), congestive heart failure (CHFx2 pts), atrial fibrillation (AF), cerebrovascular accident (CVA), and stage 3 chronic renal failure. These preoperative medical comorbidities reflect the nature of this high-risk patient population.

All patients had preoperative CT scans, MRI, transesophageal echocardiogram, and cardiac catheter studies at the time of endovascular downstaging, with physiological assessment of cardiac function of each chamber.

The median operative time was 280 min (range 224–331 min). The median intraoperative blood loss was 1875 ccs (range 400–5500 ccs). There were no intraoperative or post-operative complications. Only one patient required a one-unit transfusion postoperatively. The median length of the stay was 5.5 days (range 5–6 days). The median size of the tumor was 13 cm (range 10–17 cm). And the median Fuhrman score was three/four (Table 1).

In this series of patients undergoing endovascular downstaging, all four patients were diagnosed with metastatic disease. Two patients died of the disease, whereas one patient is alive with the disease, and one patient with multiple pulmonary metastases initially had complete, spontaneous regression of all his pulmonary nodules, as evidenced on his postoperative follow up chest CT scans and remains alive and well without evidence of recurrence.

Despite the metastatic progression of their cancer, it is remarkable that the mean overall survival of these patients following their surgery was 4.75 years, ranging from 2.5 to 8 years, as shown in Table 2. Post-operative renal function was also excellent, with a GFR of 54 or higher in three of the four patients.

## 5. Discussion

Despite significant surgical advances and substantial improvements in perioperative care, the management of patients with renal cell carcinoma and IVC level III and IV tumor thrombus remains complex and is associated with significant complications, morbidity, and mortality by the very nature of the procedures currently used [20,21,22,23,24,25].

Various techniques for the removal of intracaval tumors have been described, and their techniques have evolved over time. We initially used the standard median sternotomy CPB approach for level III and IV tumor thrombi [11,12,13,14,15]. However, we transitioned to the minimal access approach, originally described by Cosgrove for aortic valve surgery, and we modified it in 1998 for use in renal cancer cardiopulmonary bypass surgery [16,17].

The minimal access right parasternal approach to the right atrium and super hepatic vena cava has become a very useful development for the urologic surgeon for radical nephrectomy and vena cava thrombectomy. It also provides excellent exposure to all portions of the inferior vena cava if the caval wall is invaded and resection and reconstruction become necessary. Additionally, it reduces thoracic trauma, resulting from the smaller incision and the absence of a sternotomy, and facilitates pulmonary recovery, leading to earlier extubation, shorter ICU and hospital stays, and reduced wound complications. Minimal access surgery improved our clinical results compared to the median sternotomy approach we originally used [18]. Minimal access surgery, therefore, has become our preferred method of dealing with level III and IV tumor thrombi.

However, significant complications, morbidity, and mortality occur because of deep hypothermic cardiac arrest and cardiopulmonary bypass [26,27,28,29,30]. In a report from the Mayo Clinic, which reviewed their experience with renal cell cancer and IVC involvement from 1970 to 2000, complications occurred in 15% of patients undergoing this type of surgery. The study reported an increased rate of both major and minor complications proportional to the level of the tumor thrombus. For IVC tumor thrombus levels I through IV, adverse events rates were noted at 18%, 20%, 26%, and 47%, respectively. Among the most frequent complications were hemorrhage, 3%, pulmonary embolism, 2.7%, wound infection, 2.65%, acute renal failure, 1.8%, and the need for additional surgery, 3.6% [31]. A 26% complication rate for level III and a 47% complication rate for level IV tumor thrombi is formidable, especially in the hands of this very experienced, respected surgical team. Thus, there is an unmet need to reduce surgical complications and improve short-term surgical outcomes and long-term oncological outcomes for patients with RCC and IVC level III and IV thrombi.

We became interested in an alternative approach when confronted with a unique clinical situation, which precluded our use of DHCA and CPB [19]. This current group of endovascular down staged patients have been retrospectively reviewed and compared to our experiences with DHCA and CPB, both the standard median sternotomy and the minimal access approach.

The median operative time for the endovascular downstaging group is 280 min, and the median blood loss is 1875 cc. This compares favorably to the standard median sternotomy, with a median operating time of 600 min and median blood loss of 5500 cc, and to the minimal access patients, with a median operating time of 476 min and a median blood loss of 3750 cc [18]. The intraoperative results in the endovascular down staged patients, with shorter operating times and less blood loss, compares favorably to our bypass groups of patients. Only one patient in the endovascular down staged group received a single unit of blood postoperatively. The significant reduction in operative time and decreased blood loss are clinically beneficial, especially in this high-risk patient population, as noted by their preoperative medical conditions (Table 1).

As stand-alone surgical procedures, even without tumor thrombectomy, DCA and CPB are associated with a very long list of complications: seizures, decreased cognitive function, chronic renal failure, cardiac ischemia (if greater than 90 min), circulatory arrest, visual defects, coagulopathy, exaggerated inflammatory response, and activation of the complement system [26,27,28,29,30]. All these adverse side effects contribute significantly to the morbidity and mortality of DHA and CPB. Eliminating DHCA and CPB eliminates all the above-mentioned potential complications.

Downstaging also reduces the complexity of tumor thrombus surgery. Neoadjuvant regimens have not demonstrated significant benefits for patients with locally advanced kidney cancer [32,33], lending further justification and importance to this new method of endovascular tumor thrombectomy as an effective means of downstaging. A simpler operation reduces surgical complications and reduces our average length of stay (ALOS); which in the median sternotomy was 26 days, and in the minimally invasive approach was 12 days [9]. In the endovascular downstaging group, ALOS was reduced to 5 days.

The reduced operating time, reduced blood loss, and the elimination of DHCA and CPB and all its potential complications, have combined to reduce the incidence of surgical complications, which is reflected in an absence of complications and mortality in this review of patients undergoing endovascular downstaging.

Occasionally there will be IVC tumors that are adherent or invading the wall of the IVC, and downstaging cannot be achieved. If endovascular downstaging fails, these patients will have to be treated in the traditional manner, that is DHCA and CPB. In this instance, we would recommend the minimal access approach [16,17].

This initial series of patients undergoing endovascular downstaging of level III and level IV IVC tumor thrombi represents a high-risk group in terms of cancer biology, with all four patients being diagnosed with metastatic disease. Yet, it is remarkable that these patients with life-threatening tumor thrombus had no surgical or treatment-related complications following endovascular downstaging in conjunction with their surgery, with a short length of hospital stay and no significant post-operative morbidity, thereby permitting them to proceed with systemic treatment, as indicated postoperatively. Furthermore, their excellent postoperative renal function permits these patients to undergo systemic immune and targeted therapies, in which more effective regimens have become available. Perhaps this accounts for their significant overall survival following surgery, ranging from 2.5 to 8 years (mean 4.75 years), with two of four (50%) patients surviving at least 5 years or longer following surgery. While a direct comparison of this early experience of endovascular downstaging to the robust experience with median sternotomy and minimally invasive CPB [9] is not really feasible due to the small number of cases, it is noteworthy that long-term overall survival with CPB approximates 25% beyond 5 years, which is exceeded by our present experience with endovascular downstaging. Whether this is due to the lower morbidity of surgery following endovascular downstaging, more contemporary and effective systemic therapies that have become available, or both factors remains to be determined. Nevertheless, our present experience and findings in this series of patients, with low morbidity and long-term survival following endovascular downstaging, is safe, effective, and justifies the continued and broader/expanded use of this revolutionary technique for the removal of level III and IV IVC tumor thrombi without the need for cardiopulmonary bypass.

## 6. Conclusions

Endovascular downstaging creates a new paradigm for the management of suprahepatic and supradiaphragmatic (level III and IV) IVC tumors in properly selected patients. It reduces the tumor thrombus to below the hepatic veins, making the subsequent radical nephrectomy and tumor thrombectomy less technically difficult, which results in shorter operative times, less blood loss, fewer surgical complications, lower morbidity and mortality rates, and results in shorter lengths of stay. Most importantly, it also eliminates DHCA and CPB and all of its inherent complications, which also significantly contributes to the major reduction in morbidity and mortality associated with these procedures. This proof-of-concept study demonstrates the safety and efficacy of endovascular downstaging.

In addition, it allows for this life-saving surgery to be carried out in medical centers or hospitals where cardiac surgery is unavailable, or when cardiopulmonary bypass is medically contraindicated.

## Figures and Tables

**Table 1:** Patient demographics.

	NK	DV	RS	LF
Age	54	64	74	84
Gender	M	M	M	F
Side of Tumor	R	R		L
Pre-Op Med	Massive PE, MI	MI, Mult Pulm. Mets	CHF, PE	AF, CVA, CHF,
Cond				CRF Stage 3
Op Time	224 min	300 min	264 min	331 min
Median 280 min				
Blood Loss	400 cc	1750 cc	5500 cc	2000 cc
Median 1875 cc				
# Units Post-Op	0	0	1	0
Transfusions				
Surgical	0	0	0	0
Complications				
LOS	5	6	6	5
Level of Thrombus	IV	III	IV	III
Tumor Size (cm)	17 × 9.5 × 8	9 × 9 × 9	15 × 11 × 9	10 × 7 × 5
Fuhrman Score	3/4	3/4	3/4	3/4
One Thru Four				

**Table 2:** Long-term follow up: survival and renal function.

	NK	DV	RS	LF
Surgery	4/17/15	7/3/19	1/16/20	3/16/2021
First recur	6/19/15	Pulm Mets @surg	9/16/20	+LN@ surg
Last f/u	6/25/23	6/24/24	6/16/22	5/30/24
Status	DOD	NED	DOD	AWD
DFS	2 mos	5 yrs	8 mos	+LN@surg
OS	8 yrs	5 yrs	2 yrs 5 mo	3 yrs 2 mo
Serum Cr	0.9	1.4	1.1	1.2
GFR	>60	54.4	>60	41
Date	6/20/15	5/31/24	6/10/22	8/10/21

## Data Availability

Data are contained within the article.
